# Psychological well-being of adolescents with physical disabilities in Zimbabwean inclusive community settings: An exploratory study

**DOI:** 10.4102/ajod.v6i0.325

**Published:** 2017-10-26

**Authors:** Jabulani Mpofu, Maximus M. Sefotho, Jacobus G. Maree

**Affiliations:** 1 Department of Disability Studies and Special Needs Education, Zimbabwe Open University, Zimbabwe; 2 Department of Educational Psychology, University of Pretoria, South Africa

## Abstract

**Background:**

The purpose of this study was to explore the psychological well-being of adolescents with physical disabilities living in inclusive community settings of Makonde Urban in Zimbabwe. An inclusive community is one that aims to remove exclusionary practices within the community and promote community systems that accept all people irrespective of their difference. Inclusive communities on their own are not uniquely designed for people with disabilities, but most developing countries have adopted them as a basic strategy to influence and enhance psychological well-being of people with disabilities.

**Methods:**

A constructivist lived experience perspective underpinned this research, in which multiple case studies were used to interact with the participants on inclusion and psychological well-being of adolescents with physical disabilities. Purposive sampling was used to select 14 participants (9 males and 5 females). Data were collected through face-to-face interviews and transcribed verbatim. Four themes emerged from the thematic analysis of data sources.

**Results:**

It was found that participants who were adolescents with physical disabilities living in inclusive community settings of Makonde Urban in Zimbabwe were having high levels of autonomy and choice, purpose in life, positive relations with others and good personal growth and self-acceptance.

**Conclusion:**

The findings of this study should enable inclusive communities’ policy-makers and researchers to better understand the psychological well-being of adolescents with physical disabilities living in inclusive communities.

## Disability inclusive communities

Disability inclusive communities are those communities that facilitate the inclusion of people with and without disabilities, rather than expecting individuals to fit into the existing arrangements (Chakuchichi & Mutamiswa 2003; WHO 2004). Inclusive communities are not about the vulnerable members of the community, but they constitute a framework through which all community development can take place (Ainscow 2003). Inclusive communities recognise that all community members, not only those who are vulnerable to marginalisation, require communities that are responsive to all aspects of community diversity (Ainscow 2003). The core value of an inclusive community is acknowledging that people are different and that diversity should be valued (Chakuchichi, Chimedza & Chinze 2003; Engelbrecht & Green 2007). An inclusive community engages in both sustained and sustainable strategies that are designed to address the needs of its diverse people, including those with disabilities.

## Disability inclusivity in developing country settings

The general quality of life for people with disabilities in developing countries has shown some improvement over the last decade. This has been largely because of the involvement of these individuals with disabilities in various inclusive community strategies (Choruma 2006). The adoption of inclusive community practices by many developing countries has directly influenced the movement of most adolescents with physical disabilities from disability group homes to living in more inclusive communities (Majoko 2005; Makuyana 2004; Mkandla & Matarutse 2002). Those who remained behind in group homes are catered for by special institutions and residential rehabilitation hospitals that are scattered in many developing countries (Hungwe 2005; Mpofu & Shumba 2013; Mpofu et al. 2012), as inclusion has its own limits (Hansen 2012). Other adolescents with physical disabilities are kept indoors by their parents for various reasons, including attitude-related reasons (Choruma 2006). An example of an attitude-related reason would be when family members consider their children with physical disabilities as incapable of socialising with others (Choruma 2006; Mpofu 2003).

People with disabilities tend to be less well accepted by the majority of people in some societies (Ndawi 2000). The rejection of minority status groups is described in terms of stigmatisation (Lazowski et al. 2012). Many people who share mainstream cultural values stigmatise persons with disabilities, making statements about attributes of people with disabilities that are deeply discrediting (Mpofu & Harley 2000). They consider people with disabilities as less than fully human (Corrigan & Watson 2002; Martz 2004). These attributes could be visible disability, skin colour, race or geographical cultural value. However, the more visible the attribute like a physical disability, the more stigma it attracts for the beholder, and the greater disruption it can cause to social relations, or personal relations with others (Mpofu 2003).

Inclusive communities are concerned with the identification and removal of barriers to community adjustment, development and participation (Ainscow 2003). Inclusive communities have the potential to serve as the context for the creation of sustainable and free support systems and a means of communication adapted to meet the diverse needs of community members. Inclusion in community is about the presence, participation and achievement of all community members. Presence implies location, that is, where the individual is, and participation is concerned with the quality of his or her experience (Ainscow 2003). Although inclusive communities are not uniquely designed for vulnerable populations, they have been adopted by most communities as a basic strategy to influence and enhance the psychological well-being of their people with disabilities.

## The Zimbabwe situation

The Zimbabwean government adopted the policy of an inclusive community in 1997 as a measure to enhance the psychological well-being of adolescents with physical disabilities (Chakuchichi et al. 2003; Mutamiswa & Chakuchichi 2003). However, its implementation has been wrought with several challenges, some of which include the incompatibility of the programme with consumer cultures and continued negative attitudes of community members without disabilities towards those with disabilities (Chidyausiku 2000; Mpofu 2003). The inclusive communities’ programmes that are being implemented in Zimbabwe and in other developing countries support western perspectives on disability. Western perspectives on disability generally differ from African perspectives as the latter are based on local cultures (Chakuchichi & Chimedza 2003), and they operate from the broader attitudes of society (Mpofu 2003). African societies as a rule view disability as a product of sin or a curse, and efforts to address challenges emanating from such causes must therefore be directed at the family and not the community level (Chakuchichi & Chimedza 2003; Mpofu 2003; Mpofu et al. 2012). This view is, however, not peculiar only to African cultures. It is also found in a wide range of religions, including those rooted in western societies. For example, from a Christian perspective, the Bible makes numerous references to diseases and disabilities as punishment from God for immoral acts, and efforts to address these challenges include repenting and having faith in God (Deut. 27:27; John 5:14; Matt. 9:2).

Zimbabwe currently has no legislation for inclusive communities (Choruma 2006; Mpofu et al. 2007). The operation of inclusive communities in the country is governed by policies instead of laws. Zimbabwean inclusive policies are captured in the form of circulars that give guidance in inclusive communities in Zimbabwe and are designed at department level. An example of such a policy is the Director Circular No 3 of 2001, which contains guidelines on providing equal access to education for learners with disabilities (Chakuchichi & Mutamiswa 2003). Laws on inclusive communities’ services are necessary for the funding and accountability of these programmes (Chakuchichi, Chimedza & Chinze 2003; Mutamiswa & Chakuchichi 2003). In the absence of any mandatory order stipulating the services to be provided, there can be no meaningful inclusive community services for adolescents with physical disabilities in Zimbabwe. The absence of inclusive community laws in Zimbabwe demonstrates that the country is not yet ready for the costs related to inclusive practices (Mpofu et al. 2012a, 2012b). In fact, according to Choruma (2006) and Mpofu (2003), the country delegated the costs related to caring for people with physical disabilities to already poor communities. Inadequate policies on the funding of inclusive community activities are evidenced in the country’s constitution. For example, Section 83 of the Zimbabwean Constitution, which deals with the Rights of Persons with Disabilities, limits the provision of services and resources by the state to people with disabilities (COPAC 2013).

## Psychological well-being with disability

Psychological well-being is a multifaceted concept (Kahneman & Krueger 2006). It is generally believed that psychological well-being is made up of five key psychological aspects. These are autonomy and choice, having a purpose in life, positive relations with others, personal growth and self-acceptance (Dolan, Layard & Metcalfe 2011; Kahneman & Deaton 2010). Autonomy and choice involve displaying qualities such as self-determination, independence and regulation of behaviour from within (Desmarais & Savoie 2011; Fredrickson 2001). Adolescents with physical disabilities who are actualising their potential are described as exhibiting autonomous functioning and resistance to enculturation (Laumann et al. 2006). They are also described as having an adequate internal locus of evaluation, that is, they do not need to look to others for approval but evaluate themselves by personal standards (Fredrickson 2001). Purpose of life refers to having beliefs that give adolescents with physical disabilities feelings that there is purpose in and meaning to life (Dolan et al. 2011; Kahneman & Deaton 2010). Adolescents with physical disabilities should also be expected to have varieties of changing purposes or goals in life such as being productive and creative, or achieving emotional integration later in life. Positive relationships with others include the ability to love and are a central component of mental health (Diener et al. 2009; Kahneman & Krueger 2006). Self-actualising adolescents with physical disabilities are described as having strong feelings of empathy and affection and are further described as being capable of greater love, deeper friendship and more complete identification with others (Diener et al. 2009). Relating to others with warmth is posed as a criterion of maturity. Adult development stage theorists (Freud, Erickson) support this view by emphasising the achievement of close unions with others and guidance and direction of others as a criterion of maturity.

Personal growth involves enhanced cognitive function among adolescents with physical disabilities, and confronting new challenges or tasks at different periods of life successfully (Dolan et al. 2011; Kahneman & Deaton 2010). Self-acceptance refers to a central feature of mental health as well as a characteristic of self-actualisation and optimal functioning and maturity (Kahneman & Krueger 2006). Holding positive attitudes towards oneself by adolescents with physical disabilities thus emerges as a central characteristic of psychological well-being. This enhances self-acceptance by adolescents with physical disabilities – a central characteristic of psychological well-being (Diener, Lucas & Oshi 2002).

## Goal of the study

This study aimed to explore the psychological well-being of adolescents with physical disabilities living in inclusive community settings of Makonde Urban in Zimbabwe. We specifically aimed to facilitate accessing marginalised experiences and voices of adolescents with physical disabilities.

## Method

### Research design

This study was guided by a constructivist perspective (Braun & Clark 2006; Creswell 2012) and by the principles of thematic analysis (Braun & Clark 2006; Creswell 2009, 2012). Given that the aim of the study was to explore the psychological well-being of adolescents with physical disabilities, a phenomenological research approach in which adolescents with physical disabilities experiences and voices are foreground in both design and analysis was appropriate.

### 6.2. Sampling and sampling techniques

The purposively selected sample for this study comprised nine male and five female adolescents with physical disabilities. The sample enabled the researchers to collect adequate (rich) data for the study (Cohen, Kahn & Steeves 2000; Creswell 2007). To be included in this study, the adolescents must have been staying in inclusive communities of Makonde Urban continuously for a period of not less than 2 months.

### Data collection

Consistent with the constructivist research perspective adopted in this study, open-ended interviews (Baxter & Jacke 2008; Scholz & Titje 2002) were used to collect data. Participants responded to one-on-one interview questions based on a prepared interview schedule on how adolescents with physical disabilities construct their psychosocial well-being experiences in inclusive communities. The interviews were recorded using a mobile phone, with each interview lasting between 60 and 90 minutes. The notes and audio-tape recordings were used for the purpose of this study only, and they will not be made available to any other person or to organisations not involved in this study. The interviews were conducted, transcribed and analysed by the first author.

### Data analysis strategies

Data were analysed following the thematic content analysis approach proposed by Grbich (2004): becoming familiar with the data; transcribing of the interviews; creating codes linked to research questions by identifying key words and sentences; grouping codes into themes and the last stage involved reviewing themes, labelling them and selecting appropriate quotes to represent the themes. To enhance the dependability of our study, we allowed peer debriefing in this study in order to see agreement in data labels and the logical paths taken to arrive at those labels. In addition, we also ensured confirmability of the findings by making an audit trail to our study and authors reflexivity. We also provided thick description throughout our study to check for the transferability of our study.

### Ethical consideration

Ethical approval reference number EP 15/04/01 for the study was obtained from the Ethics Committee of the University of Pretoria. Standard ethical principles of informed consent and voluntary participation, protection from harm, confidentiality and privacy were adhered to throughout the research process and of data collection and analysis. Assurance was given that no person would be identified.

## Findings and discussion

The findings of this study are divided into four main sections: (1) autonomy and choice in life, (2) purpose of life, (3) positive relations with others and (4) personal growth and self-acceptance. These are the major attributes of psychological well-being identified by this study.

### Autonomy and choice in life

Most participants (10 out of 14; 71%) in this study indicated that their participation in inclusive communities’ activities was contributing the development of their autonomy and choice. Below, we present the verbatim data from the participants. Chiwaridzo (pseudonyms are used) had this to say on autonomy:

‘Because I am making progress at my university I have a feeling that I am being empowered. This empowerment is giving me some feelings of self-rule and choice. I selected subjects to take at advanced level and selected accounting at the costs of so many other programmes in the faculty of commerce. This choice is coming all as a result of learning.’ (Participant 6, male, 19 years, myelodysplasia)

Another participant, Jinye, said:

‘Through using computers, I feel I am being equipped with abilities to make choices. Games such as chess are all about making correct moves. If you move wrongly you are defeated so learning through games makes me develop making right choices not only in the game of chess but in life.’ (Participant 7, male, 16 years, hemimelia)

However, Nyarai had a different perspective on how learning was influencing her autonomy and choice. She said:

‘Learning was not doing enough to enhance my feeling of control over my choices. Most of the materials I learn and do in my life is controlled by my parents and teachers my brothers, sisters and even friends. I am always told this is what I can do and I can’t do.’ (Participant 3, female, 15 years, limb deficiency)

Dimingu said:

‘I do not have choice in my life my health condition is giving me limitations. I cannot even write a lot of school work or play with others for a long time because I easily get tired even if I want to.’ (Participant 8, female, 15 years, epilepsy)

Jeff also said:

‘Through play my social needs are addressed at group level. People need to be happy and this is possible when they have choices around them and if their choices are accepted by others they share time with. In my case I have visitors who come to our school and talk with us about our needs. We tell them some of our needs are addressed but most of them are not. However, we would have given out our choices.’ (Participant 5, male, 15 years, traumatic brain injury)

Nyarai said:

‘The fact that I do not have my left leg controls me in choosing what I want to do in my community as long as it requires the use of the missing leg. What I am saying is my disability affects my choice.’ (Participant 3, female, 15 years, limb deficiency)

Dimingu added:

‘I am not developing any choice. The choice is determined by my health. I have epilepsy it attacks me without notice and because of that you cannot just do anything you want my choice is limited.’ (Participant 8, female, 15 years, epilepsy)

Our finding that adolescents with physical disabilities living in inclusive community settings tend to display good levels of autonomy is consistent with findings from Gabre (2000) and Gabre et al. (2002). Gabre (2000) and Gabre et al. (2002) asserted that less restrictive living arrangements for people with disabilities like living in inclusive communities led to increased prevalence of feelings of autonomy. These authors explain that these are positive developments because it means that people with disabilities living in inclusive communities tend to feel similar to their peers. Conversely, Emerson et al. (2009) found out that people in the United Kingdom (UK) who require ongoing support with daily living tasks (common to people with physical disabilities) and have very limited verbal communication (e.g. those with severe cerebral palsy) are unable to express their opinions and preferences and therefore rely on others to help them take important decisions. Mpofu and Shumba (2013) also found out that people with disabilities in Zimbabwe were not being given an opportunity to talk about their health problems when they visit doctors. Instead, their ‘substitute decision-makers’ were responsible for making decisions on their behalf, including when to go to the clinic. In this study (Mpofu & Shumba 2012), participants indicated that they were being stereotyped as incompetent patients, unable to present or articulate their health care needs.

### Purpose of life

Twelve out of fourteen participants (85%) in this study said that their participation in inclusive community activities was helping them to develop feelings of having a sense of purpose in life. For instance, Chiwaridzo had this to say on purpose of life:

‘I am satisfied with the progress I have made in my life. I am doing well more than what other people of my age without disabilities do. It’s an achievement to be at a university considering that I have a disability. I am satisfied and have no problems.’ (Participant 6, male, 19 years, myelodysplasia)

Walter had a different view on learning. He said:

‘The learning I am getting is unsatisfactory. I am being taught things that are not of much benefit to my life. I want to be a truck driver. I admire my uncle who drives a truck. Instead of being taught math’s English and others which takes a lot of time to be employed they must teach me driving.’ (Participant 10, male, 14 years, spinal cord injury)

Jinye said:

‘I was going to be satisfied with my life if I was learning carpentry. The learning I am getting both at school will never give me the life I want to live. I want to be a carpenter.’ (Participant 7, male, 16 years, hemimelia)

Kombo has this to say on purpose of life:

‘When I participate in social issues with my peers with and without disabilities I get happy. I also get happy when I am consulted over my life.’ (Participant 11, female, 13 years, polio)

Try said:

‘I learn more about my condition at home from my parents, others who live with HIV like me. These people are very important. They have made me understand my condition and that I can live a normal life like any other person living without HIV. They have made me understand that I am a person like any other person in my community. They have made me know that what is.’ (Participant 13, female, 15 years, HIV positive)

This study’s findings are similar to the findings of Nygren et al. (2005), who found that people with disabilities in the UK who live in inclusive community settings achieved high scores on the *Perceived Purpose in Life Test* (Kahneman & Deaton 2010) and the *Self-transcendence Scale* (Dolan et al. 2011). Although this finding is consistent with Nygren et al.’s (2005) finding, Albrecht and Devlieger (1999) suggest that it is not purpose of life per se that is enhanced but, instead, blissful feelings about a real purpose in life. Albrecht and Devlieger (1999) built on the work of So Levine to examine the following disability paradox: People with serious and persistent disabilities living in inclusive communities often report that they experience a keen sense of purpose of life when, to most external observers, these individuals seem to experience an undesirable daily existence. Closer analysis of Albrecht and Devlieger’s (1999) interviews revealed that some of the participants indicated that they were in fact not expecting a brighter future.

### Positive relations with others

Eight out of fourteen participants (57%) in this study reported to have good relationships with their peers, evidenced by the following examples.

Langton said:

‘As learners, we are always a family be it at school or home. I have friends, I belong to group C, I am in grade 4 we wear a blue uniform the whole school. When we are at sports we support one team our school and if we win we all get happy if we lose we all get sad.’ (Participant 2, male, 14 years, neuromuscular disorders)

Another participant, Try, said:

‘We learn in groups be it at home or school. At my home, our parents teach us good manners. If we behave well as a family, they become happy. If one of us do something bad our parents gets angry. They teach us how to do well before visitors and where ever we are. We also help each other by telling ourselves to do well as people belonging to our family.’ (Participant 13, female, 15 years, HIV positive)

Kevie said:

‘My involvement in so many issues with my friends and being consulted on issues around my disabilities makes me feel that I belong to the same group as others without disabilities in my community.’ (Participant 1, male, 18 years, cerebral palsy)

Kombo said:

‘I have learnt to respect my friends through playing with them in my community. Of course, we learn it at school at assemblies, social studies and Religious studies but we do it out of school. You see that you are doing it well mostly when you are not in class.’ (Participant 11, female, 13 years, polio)

The finding that living in an inclusive community enhances development of their positive relations with others among adolescents with physical disabilities is consistent to research findings by Magiati, Dockrell and Logotheti (2002). These authors carried a study on young Greek children’s understanding of disabilities in terms of the influence of development, context and cognition in children’s representations of different disabilities. Seventy-nine 8- to 9-year-old and 10- to 11-year-old Greek children with disabilities living in inclusive settings were involved in the study to see if they were selecting friends based on individual differences such as disabilities. Children from an urban school and rural communities were involved in the study. In their responses to interviews on their attitude on inclusion, they provided generally positive views of inclusion (Magiati et al. 2002). However, children were less positive about activities that might directly reflect upon themselves. Children’s responses in the interviews indicated that they were developing rich representations of differences and diversities. Children had the greatest understanding of sensory and physical disabilities and indicated that living in inclusive communities was improving their relations with peers with and without disabilities.

Positive relationships with others are very important for successful adjustment and integration of people with disabilities. Experiencing positive relations with others also plays an important role in facilitating social and moral development of human beings (Heward 2003). This explains that being in an inclusive community is not just enough to promote positive relations with others for people with physical disabilities, what really matters is whether people exhibit their acceptance for people with disabilities.

### Personal growth and self-acceptance

Personal growth was an inclusive community quality endorsed by all participants (100%).

On personal growth and self-acceptance, Chiwaridzo said:

‘Because of being engaged in learning I feel I am growing well. I can now do a number of things by myself. I can read, write and am adding value to myself very soon I will be an accountant.’ (Participant 6, male, 19 years, myelodysplasia)

Langton also said:

‘Going to school makes me learn so many things. Learning so many things this shows that I am growing. I was once in grade one but I am now in grade four. I have changed so many teachers and all my teachers are saying well you are growing well. Look I am now able to do so many things alone at home things that I was not able to do before because of my condition. I am now seeing that I am not disabled. I am growing up.’ (Participant 2, male, 13 years, neuromuscular disorder)

Try said:

‘Sport is assisting me growing all-round. It helps me to play well with others although it is painful when you lose. But it helps me a lot. My participation in sport makes me feel that I am being recognised at school as a person who is not disabled but abled.’ (Participant 12, female, 14 years, HIV positive)

Another participant, Jinye, said:

‘In chess you win, lose or draw. These are the results awaiting any chess player so when I win like in most of my cases I felt great and my supports will value me more. At my school if I win or lose my head always see well in me. I will be put in front of other students on the assembly and the students will be asked to clap hands and cheer for my results. Besides that, if I win at school, level. Win again with other schools that movement from one stage to another makes me feel growing in my sport.’ (Participant 7, male, 16 years, hemimelia)

Our finding is consistent with Gallant’s (2003) findings on the influence of Social Support systems such as inclusive communities on chronic illness self-management. Gallant (2003) reviewed empirical literature examining the relationship between inclusive communities and chronic illness and self-acceptance. He identified 29 articles, of which 22 were quantitative and 7 were qualitative. The majority of research in this area concerns diabetes self-management, with a few studies examining asthma, heart disease and epilepsy management. Taken together, these studies provide evidence for a modest positive relationship between living in inclusive communities and chronic illness self-acceptance, especially for people with diabetes. Dietary behaviour appears to be particularly susceptible to social influences. In addition, being a member of a social network has a potentially important influence on self-acceptance. It seems that there is a need to elucidate the underlying mechanisms by which inclusive community settings influence self-acceptance and to examine whether this relationship varies by disability, type of support and behaviour. There is also a need to understand how the social environment may influence self-acceptance in ways other than engaging people with physical disabilities in various physical activities in their communities

### Limitations of the study

One limitation of this study is that this study’s sample was culturally homogenous. The majority of the participants in this study were adolescents with physical disabilities in primary school education (93%). As such, the participants of this study only represent a restricted range of social experiences, and therefore, the findings may not accurately represent the experiences of those from culturally and linguistically diverse backgrounds (Baum 2003). For instance, an adolescent with a physical disability from secondary school level, or university level and non-Shona speaking western culture may hold different meanings of inclusive community activities and feelings, which may have implications for how they respond to community strategies that invite them to participate in community activities. Research that includes participants with socio-economic diversity should help to clarify the role of social class in choices and participation in inclusive communities’ activities.

### Recommendations

Based on the complex nature of the interaction between aspects such as inclusion, disability, psychological well-being and public policy, several recommendations can be made for populations with similar characteristics as the one covered by this study. This study recommends the need for further research on inclusion, disability and psychological well-being. Discourse analysis that investigates the relationship between inclusion and psychological well-being of adolescents with disabilities could lead to improved implementation of inclusion. The findings of such studies could guide the development of inclusive policies that encourage community participation of non-dominant cultures such as people with disabilities in designing community activities that enhance their personal development.

## Conclusion

The psychology of well-being is devoted to promote our understanding of the biopsychosocial and behavioural factors that contribute to enhanced well-being, optimal emotional processing and the prevention of psychological dysfunction. The overall result of this study, namely that participation in inclusive communities by adolescents with physical disabilities is particularly conducive to the development of their psychological well-being, adds to the body of knowledge in the field of psychological well-being of people with disabilities.
